# Biomarker patterns across skin–lung–gut burden in systemic sclerosis

**DOI:** 10.3389/fimmu.2026.1841944

**Published:** 2026-07-17

**Authors:** Huichen Luo, Danhui Hu

**Affiliations:** 1 Department of Rheumatology and Immunology, The First Affiliated Hospital of Guangxi Medical University, Nanning, Guangxi, China; 2 Department of Rheumatology and Immunology, The First Affiliated Hospital of University of South China, Hengyang, Hunan, China; 3 Department of Neonatology, The First Affiliated Hospital of University of South China, Hengyang, Hunan, China

**Keywords:** biomarkers, gastrointestinal involvement, interstitial lung disease, phenotype, systemic sclerosis

## Abstract

**Background:**

Systemic sclerosis (SSc) is a multisystem autoimmune disease in which skin, lung, and gastrointestinal involvement often co-occur. Whether increasing organ co-involvement burden is accompanied by coherent circulating biomarker changes remains unclear.

**Objectives:**

To characterize immune, inflammatory, and nutritional biomarker patterns across skin–lung–gut burden groups in SSc.

**Methods:**

We conducted a retrospective cross-sectional study of 314 patients with SSc fulfilling the 2013 ACR/EULAR classification criteria. Three prespecified axes were used to define organ burden: skin axis, modified Rodnan skin score (mRSS) ≥14; lung axis, interstitial lung disease (ILD); and gut axis, broad gastrointestinal involvement (bGI). Patients were classified into four mutually exclusive groups according to the number of involved axes: 0-Axis, 1-Axis, 2-Axis, and 3-Axis. Clinical characteristics and biomarker distributions were compared across groups. Multinomial logistic regression was performed using 0-Axis as the reference group, with prespecified sensitivity analyses including ordinal and treatment-adjusted models, subtype-informed analyses, and axis-specific adjusted analyses. Unsupervised clustering was used as an exploratory complementary analysis.

**Results:**

Among 314 patients, 37 (11.8%) were classified as 0-Axis, 136 (43.3%) as 1-Axis, 118 (37.6%) as 2-Axis, and 23 (7.3%) as 3-Axis. Anti-Scl-70 positivity increased across burden groups, from 51.4% in the 0-Axis group to 71.9% in the 1-Axis group, 83.1% in the 2-Axis group, and 91.3% in the 3-Axis group (p<0.001). hs-CRP differed across groups and was higher in the 2-Axis group than in the 1-Axis group (9.60 [3.13–19.40] vs 4.50 [1.79–12.03] mg/L, p=0.012 overall). Albumin decreased across groups and was lower in the 3-Axis group than in the 0-Axis group (31.70 [27.10–33.60] vs 34.30 [31.80–37.50] g/L, p=0.005 overall). In adjusted multinomial joint models, Anti-Scl-70 positivity was associated with 2-Axis versus 0-Axis (OR 3.86, 95% CI 1.46–10.22, p=0.007) and 3-Axis versus 0-Axis (OR 12.78, 95% CI 1.86–87.91, p=0.010). Lower albumin was associated with 2-Axis versus 0-Axis (OR 0.52, 95% CI 0.31–0.89, p=0.017) and 3-Axis versus 0-Axis (OR 0.38, 95% CI 0.18–0.79, p=0.009). Higher total cholesterol was associated with 3-Axis versus 0-Axis (OR 2.81, 95% CI 1.11–7.13, p=0.030). hs-CRP showed an inverse association for 1-Axis versus 0-Axis (OR 0.60, 95% CI 0.37–0.98, p=0.039), but was not associated with 2-Axis versus 0-Axis (OR 0.66, 95% CI 0.42–1.04, p=0.074) or 3-Axis versus 0-Axis (OR 1.04, 95% CI 0.60–1.81, p=0.890) after joint adjustment. Unsupervised clustering identified three biomarker-defined clusters with differing centroid profiles and differing axis-burden distributions. Supplementary analyses supported the robustness of the principal Anti-Scl-70 and albumin signals while clarifying the contributions of subtype structure, lung-axis predominance, treatment exposure, and GI heterogeneity.

**Conclusion:**

Increasing skin–lung–gut co-involvement burden in SSc was associated with structured circulating biomarker patterning. The most consistent adjusted signals were Anti-Scl-70 positivity and lower albumin in higher burden groups. These findings support a clinically anchored, biomarker-informed perspective on multisystem involvement in SSc, while remaining complementary to conventional subtype-based classification and requiring cautious interpretation.

## Graphical Abstract

**Figure:**
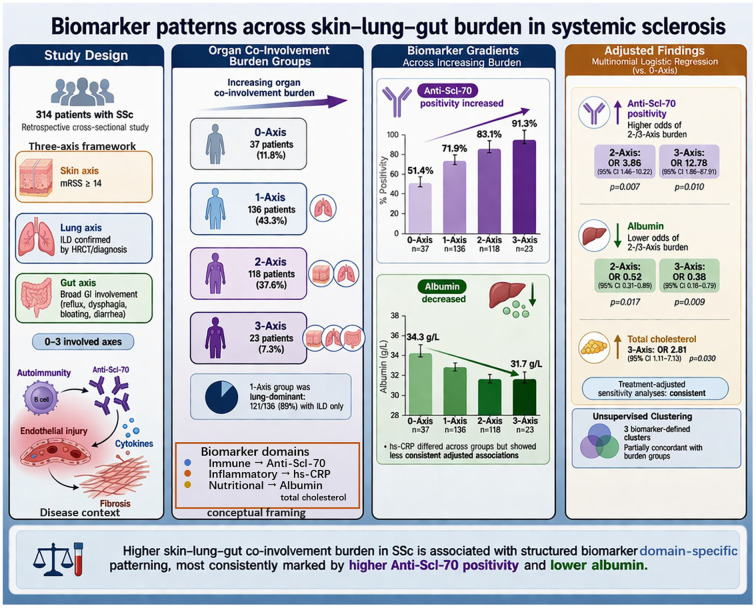

## Highlights

- A skin–lung–gut burden framework identified clinically distinct organ co-involvement groups in systemic sclerosis.
- Higher burden groups showed non-random biomarker patterning across immune, inflammatory, and nutritional domains.
- In adjusted models, Anti-Scl-70 positivity and lower albumin showed the most consistent associations with higher burden groups.
- Unsupervised clustering revealed partially concordant biomarker-defined subgroups, supporting structured heterogeneity beyond organ counting alone.

## Introduction

Systemic sclerosis (SSc) is a chronic autoimmune disease characterized by immune dysregulation, vasculopathy, and progressive fibrosis across multiple organs (1–4). Although these processes are mechanistically intertwined, clinical practice still often approaches SSc through single-organ lenses. Skin thickening remains a defining feature, interstitial lung disease (ILD) is a major determinant of long-term outcome, and gastrointestinal involvement contributes importantly to symptom burden, nutritional compromise, and reduced quality of life (2, 5–8). In many patients, however, these manifestations coexist rather than occur in isolation.

This pattern of co-occurrence is clinically important. Patients with diffuse cutaneous disease often show more extensive internal organ involvement, and serological subtype, inflammatory activity, and nutritional status may vary across clinical phenotypes (9–13). Yet studies frequently focus on one organ system or one biomarker family at a time. As a result, it remains unclear whether increasing burden across major organ domains corresponds to recognizable circulating biomarker patterns that are both clinically interpretable and biologically plausible.

Several routinely available biomarkers may be informative in this context. Autoantibody profiles, particularly Anti-Scl-70 and ACA, are linked to disease subsets and organ-risk patterns (14, 15). Acute-phase reactants and blood-cell indices may reflect inflammatory activation in selected patients (16, 17). Albumin and lipid-related markers are less disease-specific, but they may capture combined effects of chronic inflammation, gastrointestinal burden, and nutritional reserve (18, 19). These markers are widely measured in clinical practice, making them attractive for phenotype-oriented analysis. At the same time, clinical burden grouping may not fully reflect latent biological structure, so unsupervised clustering can provide an exploratory complementary view (20).

In this study, we focused on a prespecified skin–lung–gut framework. These three domains were selected because they represent common and clinically meaningful manifestations in SSc, frequently co-occur in routine practice, and can be defined in a relatively pragmatic and reproducible manner in retrospective clinical datasets. Our aim was not to construct a comprehensive severity index for all SSc organ complications, nor to replace conventional subtype-based classification, but rather to test whether increasing burden across these major co-involvement domains is accompanied by coherent biomarker patterning in a clinically pragmatic framework. The study was designed as a phenotype-oriented and hypothesis-generating analysis, rather than as an attempt to define mechanistic endotypes.

## Materials and methods

### Study design and population

We conducted a retrospective cross-sectional study of patients with SSc admitted to the First Affiliated Hospital of Guangxi Medical University between December 2018 and December 2023. Eligible patients fulfilled the 2013 ACR/EULAR classification criteria for SSc (21). Duplicate admissions were excluded by retaining the first eligible admission only. Patients with active infection, malignancy, or other chronic diseases likely to substantially distort inflammatory or immune laboratory indices were excluded. The final analysis cohort included 314 patients.

### Definition of skin–lung–gut burden groups

Three prespecified organ axes were used. Skin axis: mRSS ≥14, a threshold associated with diffuse cutaneous disease and higher risk of internal organ involvement in prior studies (22, 23). Lung axis: ILD confirmed by HRCT or established clinical diagnosis (24). Gut axis: broad gastrointestinal involvement (bGI), including clinically documented dysphagia, reflux/GERD, bloating/distension, and bowel manifestations compatible with SSc-related gastrointestinal involvement (25). The bGI was based on prespecified chart-based criteria applied to contemporaneous medical-record documentation. We used a broad pragmatic definition to capture the clinically relevant gastrointestinal burden commonly encountered in SSc. Objective GI investigations and standardized GI severity grading were not systematically available in structured form across the cohort and therefore were not incorporated into the axis definition. Patients were classified into four mutually exclusive groups according to the number of involved axes: 0-Axis: no involved axis, 1-Axis: any single involved axis, 2-Axis: any two involved axes, 3-Axis: all three axes involved. This framework was intended as a pragmatic clinical grouping rather than a complete mechanistic disease taxonomy.

### Clinical and laboratory variables

Clinical variables included age, sex, ethnicity, disease duration, diffuse cutaneous SSc (dcSSc), smoking history, alcohol use, and treatment background. Treatment variables summarized in **Table 1** included glucocorticoid use, immunosuppressant use, and biologic/targeted therapy use. Glucocorticoid use was defined as any in-hospital exposure with a prednisone-equivalent dose >0 mg/day. Drug categories were not mutually exclusive. Laboratory variables were organized into three modules: Immune module: Anti-Scl-70 positivity, ACA positivity, Anti-Ro-52 positivity, IgG, IgA, and IgM, Inflammation module: ESR, hs-CRP, WBC, PLT, and NLR, Nutrition module: albumin, BMI, total cholesterol, triglycerides, and HDL-C. All biomarker measurements were obtained from the same admission used for burden classification.

**Table 1:** Baseline clinical characteristics across skin–lung–gut organ-burden groups.

Variable	Overall (N = 314)	0-axis (n=37)	1-axis (n=136)	2-axis (n=118)	3-axis (n=23)	P value
── A. Demographic Characteristics						
Age (years)	55.0 (47.2–62.0)	52.0 (46.0–58.0)	54.5 (47.0–62.0)	55.0 (49.0–63.0)	56.0 (52.0–66.0)	0.356
Female, n (%)	205 (65.3%)	15 (40.5%)	89 (65.4%)	85 (72.0%)	16 (69.6%)	0.007**
Zhuang ethnicity, n (%)	177 (56.4%)	20 (54.1%)	76 (55.9%)	67 (56.8%)	14 (60.9%)	0.964
Disease duration (months)	13.0 (6.0–48.0)	9.0 (4.0–36.0)	13.5 (6.0–48.0)	13.5 (6.0–48.0)	17.0 (8.5–48.0)	0.192
Diffuse cutaneous SSc, n (%)	213 (67.8%)	15 (40.5%)	79 (58.1%)	98 (83.1%)	21 (91.3%)	<0.001***
── B. Personal Habits						
Smoking (ever), n (%)	77 (24.5%)	14 (37.8%)	32 (23.5%)	27 (22.9%)	4 (17.4%)	0.216
Alcohol use (ever), n (%)	69 (22.0%)	15 (40.5%)	30 (22.1%)	17 (14.4%)	7 (30.4%)	0.010*
── C. Axis-Defining Variables						
mRSS score	10.0 (6.0–18.0)	6.0 (4.0–8.0)	8.0 (4.0–10.0)	18.0 (14.0–23.0)	20.0 (15.0–26.5)	<0.001***
ILD, n (%)	257 (81.8%)	0 (0.0%)	121 (89.0%)	113 (95.8%)	23 (100.0%)	<0.001***
Broad GI involvement (bGI), n (%)	59 (18.8%)	0 (0.0%)	3 (2.2%)	33 (28.0%)	23 (100.0%)	<0.001***
── D. Immunotherapy						
Glucocorticoid use, n (%)	297 (94.6%)	31 (83.8%)	130 (95.6%)	113 (95.8%)	23 (100.0%)	0.015*
Immunosuppressant use, n (%)	297 (94.6%)	31 (83.8%)	130 (95.6%)	114 (96.6%)	22 (95.7%)	0.029*
Biologic/targeted therapy use, n (%)	5 (1.6%)	1 (2.7%)	3 (2.2%)	1 (0.8%)	0 (0.0%)	0.793

Data: median (IQR) for continuous variables; n (%) for categorical variables. Skin axis: mRSS ≥ 14; Lung axis: ILD confirmed by HRCT; Gut axis (bGI): broad GI involvement (reflux, dysphagia, bloating, diarrhea, constipation, or pseudo-obstruction). Zhuang ethnicity: Zhuang nationality. dcSSc, Diffuse cutaneous SSc. mRSS, modified Rodnan skin score; ILD, interstitial lung disease.

GC use was defined as any in-hospital glucocorticoid exposure with a prednisone-equivalent dose >0 mg/day. Immunosuppressant use referred to exposure to conventional non-biologic immunosuppressive or immunomodulatory agents. Biologic/targeted therapy use included tocilizumab, rituximab, or baricitinib. Drug categories were not mutually exclusive. P value: Kruskal-Wallis (continuous) or Chi-square/Fisher (categorical). *P<0.05; **P<0.01; ***P<0.001. Bold values indicate statistical significance at the nominal levels.

### Statistical analysis

Continuous variables were summarized as median (IQR), and categorical variables as n (%). Between-group comparisons were performed using the Kruskal–Wallis test for continuous variables and the chi-squared test or Fisher’s exact test for categorical variables, as appropriate. We selected multinomial logistic regression as the primary modeling. Given the ordered nature of the burden groups, ordinal logistic regression was additionally performed as a sensitivity analysis. Additional prespecified supplementary analyses included treatment-adjusted models, subtype-adjusted and subtype-stratified models, and axis-specific adjusted logistic regression models for the skin, lung, and gut axes separately. Analyses were performed using available data for each variable. For regression analyses, patients with missing values in model-included variables were excluded from the corresponding model on a complete-case basis. Predictors were entered as either univariable module models or a multivariable joint model including the immune, inflammation, and nutrition modules simultaneously. All models were adjusted for age (per SD), female sex, and disease duration (per SD). Continuous predictors were standardized as Z-scores per SD. Odds ratios (ORs) and 95% confidence intervals (CIs) were reported using the Wald method. Benjamini–Hochberg false discovery rate correction was applied within each model family. As an exploratory complementary analysis, unsupervised clustering was performed using core biomarkers and selected clinical variables. The number of clusters was evaluated using elbow and silhouette metrics. Principal component analysis (PCA) was used for visualization. Sensitivity analyses additionally adjusting for available treatment variables recorded at the time of assessment. Where available, prednisone-equivalent glucocorticoid dose was incorporated in dose-informed supplementary models. These analyses were intended to examine the robustness of the main associations rather than to fully resolve confounding by indication. The extent of missingness for major biomarkers was limited. All analyses were performed in R version 4.4.1. P values were two-sided.

## Results

### Cohort composition and burden architecture

**Figure 1:**
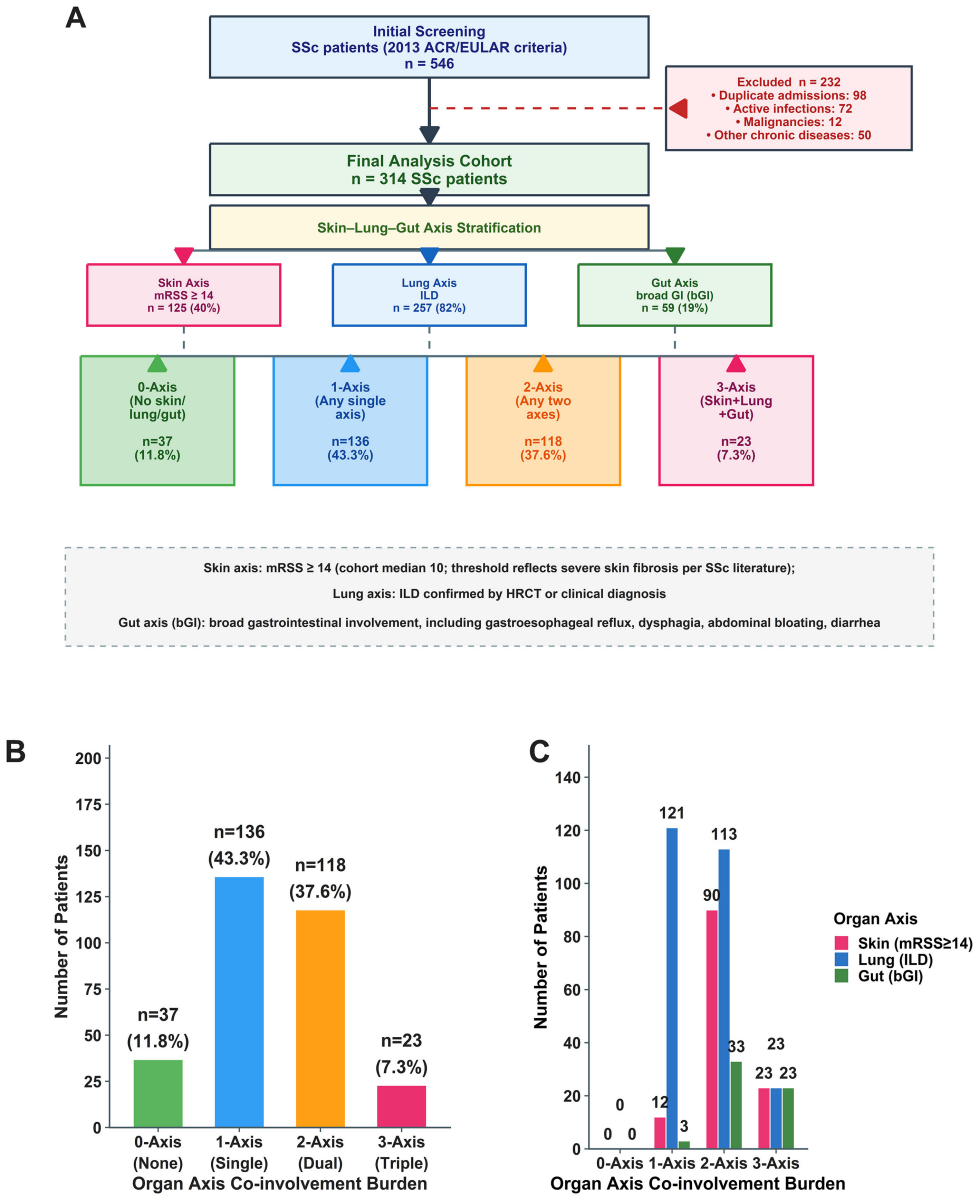
Skin–lung–gut burden framework and cohort distribution in SSc. **(A)** Study flowchart and derivation of the final analysis cohort and burden groups. **(B)** Distribution of patients across the four organ-axis co-involvement burden groups. **(C)** Internal axis composition within each burden group, showing the number of patients contributing to the skin, lung, and gut axes. ACR, American College of Rheumatology; EULAR, European League Against Rheumatism; mRSS, modified Rodnan skin score; ILD, interstitial lung disease; HRCT, high-resolution computed tomography; bGI, broad gastrointestinal involvement.

Among the 314 included patients, 37 (11.8%) were classified as 0-Axis, 136 (43.3%) as 1-Axis, 118 (37.6%) as 2-Axis, and 23 (7.3%) as 3-Axis (**Figures 1A, B**). The skin axis was present in 125 patients (40%), the lung axis in 257 (82%), and the gut axis in 59 (19%). Within the 1-Axis group, lung-axis involvement predominated, accounting for 121 of 136 patients, whereas skin-axis and gut-axis only patterns accounted for 12 and 3 patients, respectively (**Figure 1C**). Within the 2-Axis group, lung-axis counts remained frequent (113), together with skin-axis counts (90), while gut-axis counts were less frequent (33). By definition, all three axes were present in all 23 patients in the 3-Axis group. The burden was numerically dominated by lung involvement at the lower group and showed progressive enrichment of skin and gut co-involvement in the higher burden groups. This compositional structure motivated additional axis-specific adjusted analyses presented in **Supplementary Figures 6A, B**.

### Baseline clinical characteristics across burden groups

Baseline characteristics are shown in **Table 1**. Age did not differ across groups (55.0 [47.2–62.0] years overall, p=0.356), and disease duration also did not differ significantly (13.0 [6.0–48.0] months overall, p=0.192). Female sex differed across groups (p=0.007), accounting for 15/37 (40.5%) in the 0-Axis group, 89/136 (65.4%) in the 1-Axis group, 85/118 (72.0%) in the 2-Axis group, and 16/23 (69.6%) in the 3-Axis group. Zhuang ethnicity did not differ across groups (p=0.964). Diffuse cutaneous SSc differed across groups (p<0.001). It was more frequent in the 2-Axis group than in the 1-Axis group (98/118 [83.1%] vs 79/136 [58.1%]) and more frequent in the 3-Axis group than in the 0-Axis group (21/23 [91.3%] vs 15/37 [40.5%]). Alcohol use also differed across groups (p=0.010), whereas smoking history did not (p=0.216). Axis-defining variables differed as expected. mRSS differed across groups (p<0.001) and was higher in the 2-Axis group than in the 1-Axis group (18.0 [14.0–23.0] vs 8.0 [4.0–10.0]) and higher in the 3-Axis group than in the 0-Axis group (20.0 [15.0–26.5] vs 6.0 [4.0–8.0]). ILD was present in 0/37 (0.0%) patients in the 0-Axis group, 121/136 (89.0%) in the 1-Axis group, 113/118 (95.8%) in the 2-Axis group, and 23/23 (100.0%) in the 3-Axis group (p<0.001). Broad GI involvement was present in 0/37 (0.0%), 3/136 (2.2%), 33/118 (28.0%), and 23/23 (100.0%) patients, respectively (p<0.001). The internal composition of the gut-axis group is summarized in **Supplementary Table 1**.

Treatment variables also differed across groups. Glucocorticoid use occurred in 31/37 (83.8%) patients in the 0-Axis group, 130/136 (95.6%) in the 1-Axis group, 113/118 (95.8%) in the 2-Axis group, and 23/23 (100.0%) in the 3-Axis group (p=0.015). Immunosuppressant use occurred in 31/37 (83.8%), 130/136 (95.6%), 114/118 (96.6%), and 22/23 (95.7%) patients, respectively (p=0.029). Biologic/targeted therapy use was uncommon overall and did not differ significantly across groups (p=0.793). Higher burden groups were characterized by more frequent dcSSc and expected enrichment of axis-defining organ involvement.

### Biomarker distributions across burden groups

**Figure 2:**
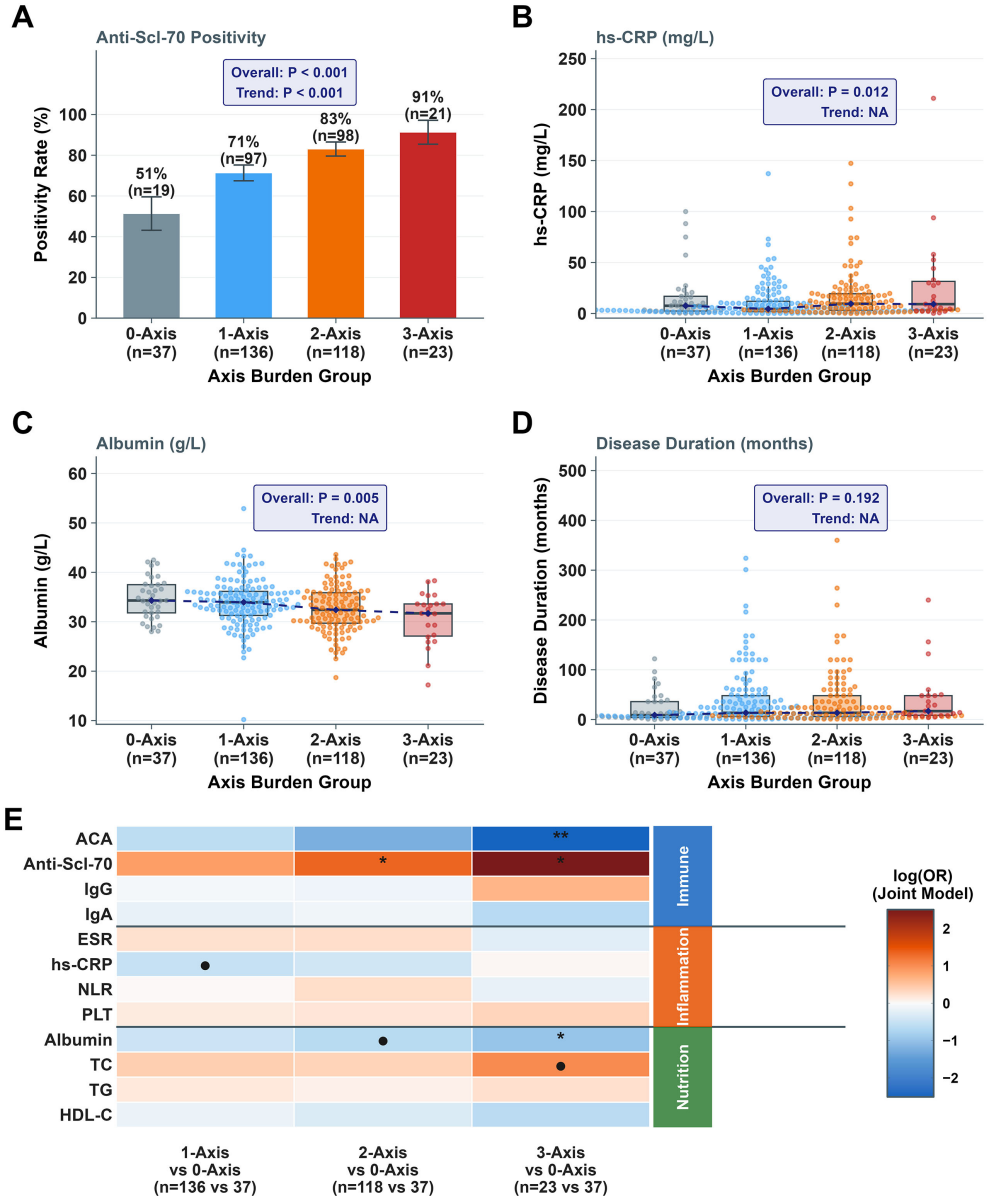
Biomarker distributions and joint-model effect patterns across skin–lung–gut burden groups. **(A)** Anti-Scl-70 positivity across 0-Axis, 1-Axis, 2-Axis, and 3-Axis groups. Bars represent proportions, and error bars indicate 95% confidence intervals. **(B)** Distribution of high-sensitivity C-reactive protein (hs-CRP) across burden groups. **(C)** Distribution of albumin across burden groups. **(D)** Distribution of disease duration across burden groups. In panels B–D, boxplots show median and interquartile range, with overlaid individual observations. **(E)** Heatmap summarizing the direction and magnitude of associations from the multivariable joint multinomial logistic regression model for selected biomarkers, with 0-Axis as the reference group. *P<0.05; **P<0.01.

Biomarker distributions are summarized in **Table 2**, **Figures 2A–D**. Within the immune module, Anti-Scl-70 positivity differed across groups (p<0.001). Anti-Scl-70 positivity was 19/37 (51.4%) in the 0-Axis group, 97/136 (71.9%) in the 1-Axis group, 98/118 (83.1%) in the 2-Axis group, and 21/23 (91.3%) in the 3-Axis group. ACA positivity also differed across groups (p=0.049), decreasing from 6/37 (16.2%) in the 0-Axis group to 15/136 (11.1%) in the 1-Axis group, 6/118 (5.1%) in the 2-Axis group, and 0/23 (0.0%) in the 3-Axis group. Anti-Ro-52 positivity did not reach statistical significance (p=0.054). IgG, IgA, and IgM did not differ significantly across groups (p=0.136, p=0.323, and p=0.849, respectively). Within the inflammation module, hs-CRP differed across groups (p=0.012). hs-CRP was lower in the 1-Axis group than in the 2-Axis group (4.50 [1.79–12.03] vs 9.60 [3.13–19.40] mg/L, p=0.012 overall), while the 3-Axis group also showed a numerically elevated median value (9.20 [3.40–31.51] mg/L). ESR did not differ significantly across groups (p=0.062), although the highest median value was observed in the 3-Axis group (39.00 [23.00–56.00] mm/h). PLT differed across groups (p=0.032) and was higher in the 3-Axis group than in the 0-Axis group (304.00 [264.80–381.00] vs 237.00 [203.90–313.80] ×10^9^/L, p=0.032 overall). NLR also differed across groups (p=0.037), increasing from 2.88 [2.29–3.87] in the 0-Axis group to 4.20 [3.14–5.58] in the 3-Axis group. WBC did not differ significantly (p=0.260). Within the nutrition module, albumin differed across groups (p=0.005). Albumin was lower in the 3-Axis group than in the 0-Axis group (31.70 [27.10–33.60] vs 34.30 [31.80–37.50] g/L, p=0.005 overall), and the 2-Axis group also showed a lower median value (32.40 [29.70–35.88] g/L). BMI differed across groups (p=0.002) and was lower in the 3-Axis group than in the 0-Axis group (19.56 [17.81–20.62] vs 21.33 [19.65–22.86] kg/m^2^). Total cholesterol, triglycerides, and HDL-C did not differ significantly in overall comparisons (p=0.160, p=0.800, and p=0.211, respectively). Higher burden groups showed more frequent Anti-Scl-70 positivity and lower albumin, together with descriptive elevations in selected inflammatory markers.

**Table 2:** Immune, inflammatory, and nutritional biomarkers across organ-burden groups.

Variable	Overall (N = 314)	0-axis (n=37)	1-axis (n=136)	2-axis (n=118)	3-axis (n=23)	P value
── A. Immune Module						
Anti-Scl-70 positivity, n (%)	235 (75.1%)	19 (51.4%)	97 (71.9%)	98 (83.1%)	21 (91.3%)	<0.001***
ACA positivity, n (%)	27 (8.6%)	6 (16.2%)	15 (11.1%)	6 (5.1%)	0 (0.0%)	0.049*
Anti-Ro-52 positivity, n (%)	79 (25.3%)	6 (16.7%)	28 (20.7%)	40 (33.9%)	5 (21.7%)	0.054
IgG (g/L)	15.57 (12.18–19.25)	14.19 (12.18–18.74)	15.12 (11.37–18.87)	16.05 (12.41–19.61)	17.79 (13.62–22.16)	0.136
IgA (g/L)	2.53 (1.87–3.38)	2.66 (2.15–3.46)	2.37 (1.74–3.39)	2.68 (2.03–3.40)	2.41 (1.94–3.12)	0.323
IgM (g/L)	1.25 (0.82–1.86)	1.15 (0.77–2.10)	1.26 (0.77–1.73)	1.24 (0.92–1.89)	1.30 (0.92–1.89)	0.849
── B. Inflammation Module						
ESR (mm/h)	28.00 (16.00–48.75)	27.00 (14.00–46.00)	25.50 (12.00–44.25)	30.00 (19.00–52.75)	39.00 (23.00–56.00)	0.062
hs-CRP (mg/L)	6.47 (2.32–18.00)	7.60 (2.40–16.90)	4.50 (1.79–12.03)	9.60 (3.13–19.40)	9.20 (3.40–31.51)	0.012*
WBC (×10^9^/L)	7.21 (6.00–9.08)	6.99 (5.09–9.20)	7.16 (6.02–8.92)	7.31 (5.94–9.24)	7.67 (6.82–8.89)	0.260
PLT (×10^9^/L)	281.65 (224.68–357.23)	237.00 (203.90–313.80)	276.45 (223.68–333.82)	289.20 (240.18–370.75)	304.00 (264.80–381.00)	0.032*
NLR	3.38 (2.31–5.21)	2.88 (2.29–3.87)	3.05 (2.25–4.71)	3.87 (2.46–6.25)	4.20 (3.14–5.58)	0.037*
── C. Nutrition Module						
Albumin (g/L)	33.40 (30.22–36.08)	34.30 (31.80–37.50)	33.95 (31.28–36.15)	32.40 (29.70–35.88)	31.70 (27.10–33.60)	0.005**
BMI (kg/m^2^)	20.69 (18.78–22.49)	21.33 (19.65–22.86)	20.96 (19.15–23.53)	20.19 (18.38–21.85)	19.56 (17.81–20.62)	0.002**
Total cholesterol (mmol/L)	4.34 (3.71–5.09)	4.07 (3.73–4.64)	4.45 (3.73–5.41)	4.19 (3.64–5.06)	4.41 (3.89–5.29)	0.160
Triglycerides (mmol/L)	1.42 (1.03–1.92)	1.43 (1.10–1.80)	1.46 (1.07–2.02)	1.38 (1.02–1.85)	1.39 (1.18–2.00)	0.800
HDL-C (mmol/L)	1.01 (0.87–1.21)	1.04 (0.93–1.22)	1.03 (0.90–1.23)	0.98 (0.81–1.17)	1.00 (0.88–1.13)	0.211

Data: median (IQR) for continuous variables; n (%) for categorical variables. Skin axis: mRSS ≥ 14; Lung axis: ILD confirmed by HRCT; Gut axis (bGI): broad GI involvement (reflux, dysphagia, bloating, diarrhea, constipation, or pseudo-obstruction). P value: Kruskal-Wallis (continuous) or Chi-square/Fisher (categorical). *P<0.05; **P<0.01; ***P<0.001. Bold values indicate statistical significance at the nominal levels.

### Multinomial logistic regression across burden groups

Adjusted multinomial logistic regression results are shown in **Table 3**. Within the immune module, in the univariable model, Anti-Scl-70 positivity was associated with 1-Axis versus 0-Axis (OR 2.64, 95% CI 1.20–5.83, p=0.016), 2-Axis versus 0-Axis (OR 4.87, 95% CI 2.07–11.43, p<0.001), and 3-Axis versus 0-Axis (OR 15.79, 95% CI 2.68–92.96, p=0.002). In the multivariable joint model, Anti-Scl-70 positivity remained associated with 2-Axis versus 0-Axis (OR 3.86, 95% CI 1.46–10.22, p=0.007) and 3-Axis versus 0-Axis (OR 12.78, 95% CI 1.86–87.91, p=0.010), whereas the estimate for 1-Axis versus 0-Axis was attenuated (OR 2.37, 95% CI 0.96–5.87, p=0.061). ACA positivity showed OR 0.68 (95% CI 0.22–2.12, p=0.508) for 1-Axis versus 0-Axis, OR 0.32 (95% CI 0.09–1.21, p=0.095) for 2-Axis versus 0-Axis, and OR 0.00 (95% CI 0.00–0.00, p<0.001) for 3-Axis versus 0-Axis in the univariable model; corresponding estimates in the joint model were OR 0.54 (95% CI 0.15–1.93, p=0.348), OR 0.29 (95% CI 0.07–1.21, p=0.089), and OR 0.00 (95% CI 0.00–0.00, p<0.001), respectively. IgG and IgA were not independently associated with burden group in the joint model. Within the inflammation module, ESR was not associated with 1-Axis versus 0-Axis (OR 1.26, 95% CI 0.66–2.43, p=0.485), 2-Axis versus 0-Axis (OR 1.29, 95% CI 0.66–2.53, p=0.459), or 3-Axis versus 0-Axis (OR 0.78, 95% CI 0.31–1.97, p=0.598) in the joint model. hs-CRP showed an inverse association for 1-Axis versus 0-Axis (OR 0.60, 95% CI 0.37–0.98, p=0.039), but not for 2-Axis versus 0-Axis (OR 0.66, 95% CI 0.42–1.04, p=0.074) or 3-Axis versus 0-Axis (OR 1.04, 95% CI 0.60–1.81, p=0.890). NLR was not associated with 1-Axis versus 0-Axis (OR 1.02, 95% CI 0.65–1.59, p=0.944), 2-Axis versus 0-Axis (OR 1.31, 95% CI 0.84–2.02, p=0.230), or 3-Axis versus 0-Axis (OR 0.83, 95% CI 0.35–2.00, p=0.682). PLT was not associated with 1-Axis versus 0-Axis (OR 1.15, 95% CI 0.73–1.84, p=0.543), 2-Axis versus 0-Axis (OR 1.22, 95% CI 0.76–1.96, p=0.418), or 3-Axis versus 0-Axis (OR 1.42, 95% CI 0.73–2.77, p=0.308) in the joint model. Within the nutrition module, albumin showed OR 0.62 (95% CI 0.37–1.03, p=0.067) for 1-Axis versus 0-Axis, OR 0.52 (95% CI 0.31–0.89, p=0.017) for 2-Axis versus 0-Axis, and OR 0.38 (95% CI 0.18–0.79, p=0.009) for 3-Axis versus 0-Axis in the joint model. Total cholesterol showed OR 1.50 (95% CI 0.81–2.77, p=0.193) for 1-Axis versus 0-Axis, OR 1.44 (95% CI 0.76–2.72, p=0.262) for 2-Axis versus 0-Axis, and OR 2.81 (95% CI 1.11–7.13, p=0.030) for 3-Axis versus 0-Axis. TG and HDL-C were not significantly associated with burden groups in the joint model. The main associations for Anti-Scl-70 positivity and albumin were directionally consistent across supplementary sensitivity analyses, including alternative model specifications, treatment-adjusted models, subtype-informed models, and glucocorticoid dose-informed models (**Supplementary Figures 1, 2, 4, 5, 7A, B**). A global effect-size overview is provided in **Supplementary Figures 3A, B**. These findings indicate that the most consistent adjusted associations with higher burden groups were Anti-Scl-70 positivity and lower albumin, whereas inflammatory markers were less stable after joint adjustment. These principal signals should be interpreted within the context of known subtype structure, lung-axis predominance, and the nonspecific nature of albumin.

**Table 3:** Multinomial logistic regression of biomarkers across organ-burden groups.

Variable	1-axis vs 0-axis	2-axis vs 0-axis	3-axis vs 0-axis
N (Total = 314)	(n=136) vs (n=37)	(n=118) vs (n=37)	(n=23) vs (n=37)
*── Immune Module (ACA, Anti-Scl-70, IgG, IgA)*			
*Univariable model*			
ACA positivity	0.68 (0.22–2.12) p=0.508	0.32 (0.09–1.21) p=0.095	Not stably estimable†, [separation]
Anti-Scl-70 positivity	2.64 (1.20–5.83) p=0.016*	4.87 (2.07–11.43) p=<0.001***	15.79 (2.68–92.96) p=0.002**
IgG (per SD)	1.13 (0.76–1.68) p=0.559	1.18 (0.78–1.79) p=0.434	1.94 (1.08–3.49) p=0.026*
IgA (per SD)	0.79 (0.53–1.18) p=0.255	0.88 (0.59–1.33) p=0.551	0.53 (0.28–0.99) p=0.046*
*Multivariable joint model*			
ACA positivity	0.54 (0.15–1.93) p=0.348	0.29 (0.07–1.21) p=0.089	Not stably estimable†, [separation]
Anti-Scl-70 positivity	2.37 (0.96–5.87) p=0.061	3.86 (1.46–10.22) p=0.007**	12.78 (1.86–87.91) p=0.010*
IgG (per SD)	0.94 (0.54–1.61) p=0.813	0.88 (0.50–1.54) p=0.657	1.92 (0.84–4.40) p=0.122
IgA (per SD)	0.84 (0.55–1.28) p=0.418	0.90 (0.58–1.38) p=0.619	0.52 (0.25–1.08) p=0.077
*── Inflammation Module (ESR, hs-CRP, NLR, PLT)*			
*Univariable model*			
ESR (per SD)	1.17 (0.73–1.87) p=0.508	1.31 (0.82–2.08) p=0.260	1.25 (0.67–2.34) p=0.486
hs-CRP (per SD)	0.64 (0.40–1.05) p=0.077	0.80 (0.52–1.22) p=0.295	1.04 (0.63–1.72) p=0.873
NLR (per SD)	0.86 (0.60–1.25) p=0.440	1.06 (0.76–1.49) p=0.729	0.75 (0.38–1.49) p=0.413
PLT (per SD)	1.34 (0.89–2.02) p=0.165	1.52 (1.00–2.30) p=0.049*	1.65 (0.94–2.89) p=0.079
*Multivariable joint model*			
ESR (per SD)	1.26 (0.66–2.43) p=0.485	1.29 (0.66–2.53) p=0.459	0.78 (0.31–1.97) p=0.598
hs-CRP (per SD)	0.60 (0.37–0.98) p=0.039*	0.66 (0.42–1.04) p=0.074	1.04 (0.60–1.81) p=0.890
NLR (per SD)	1.02 (0.65–1.59) p=0.944	1.31 (0.84–2.02) p=0.230	0.83 (0.35–2.00) p=0.682
PLT (per SD)	1.15 (0.73–1.84) p=0.543	1.22 (0.76–1.96) p=0.418	1.42 (0.73–2.77) p=0.308
*── Nutrition Module (Albumin, TC, TG, HDL-C)*			
*Univariable model*			
Albumin (per SD)	0.62 (0.39–0.98) p=0.042*	0.50 (0.31–0.82) p=0.005**	0.36 (0.19–0.66) p=0.001**
TC (per SD)	1.53 (0.88–2.64) p=0.131	1.49 (0.85–2.63) p=0.163	2.32 (1.04–5.15) p=0.039*
TG (per SD)	1.22 (0.73–2.05) p=0.449	1.08 (0.63–1.85) p=0.775	1.14 (0.59–2.21) p=0.702
HDL-C (per SD)	0.89 (0.55–1.42) p=0.616	0.74 (0.45–1.22) p=0.246	0.51 (0.22–1.15) p=0.104
*Multivariable joint model*			
Albumin (per SD)	0.62 (0.37–1.03) p=0.067	0.52 (0.31–0.89) p=0.017*	0.38 (0.18–0.79) p=0.009**
TC (per SD)	1.50 (0.81–2.77) p=0.193	1.44 (0.76–2.72) p=0.262	2.81 (1.11–7.13) p=0.030*
TG (per SD)	1.18 (0.69–2.01) p=0.544	1.09 (0.63–1.91) p=0.753	1.27 (0.62–2.61) p=0.511
HDL-C (per SD)	0.86 (0.53–1.41) p=0.550	0.73 (0.43–1.24) p=0.241	0.53 (0.20–1.38) p=0.193

OR, odds ratio; 95% CI (Wald method). Reference: 0-Axis. Covariates: Age (per SD), Female sex, Disease duration (per SD). Continuous predictors: Z-score standardized (per SD). Binary: ACA and Anti-Scl-70 (1=positive). Univariable: each module separately; Joint: all three modules simultaneously. *P<0.05; **P<0.01; ***P<0.001. † Complete or quasi-complete separation was detected; Firth penalized logistic regression was used. Bold values indicate statistical significance at the nominal levels.

### Heatmap overview of joint-model effect patterns

**Figure 2E** summarizes the direction and magnitude of joint-model associations across burden comparisons. Positive effect patterns were concentrated in Anti-Scl-70 for 2-Axis and 3-Axis comparisons, whereas negative effect patterns were concentrated in albumin across higher burden groups. Inflammatory markers showed more heterogeneous directions, with hs-CRP contributing an inverse signal for 1-Axis versus 0-Axis but not showing a consistent positive pattern for higher burden groups. This overview was concordant with the main regression results and with the broader supplementary effect-size summary (**Supplementary Figures 3A, B**).

### Unsupervised clustering analysis

**Figure 3:**
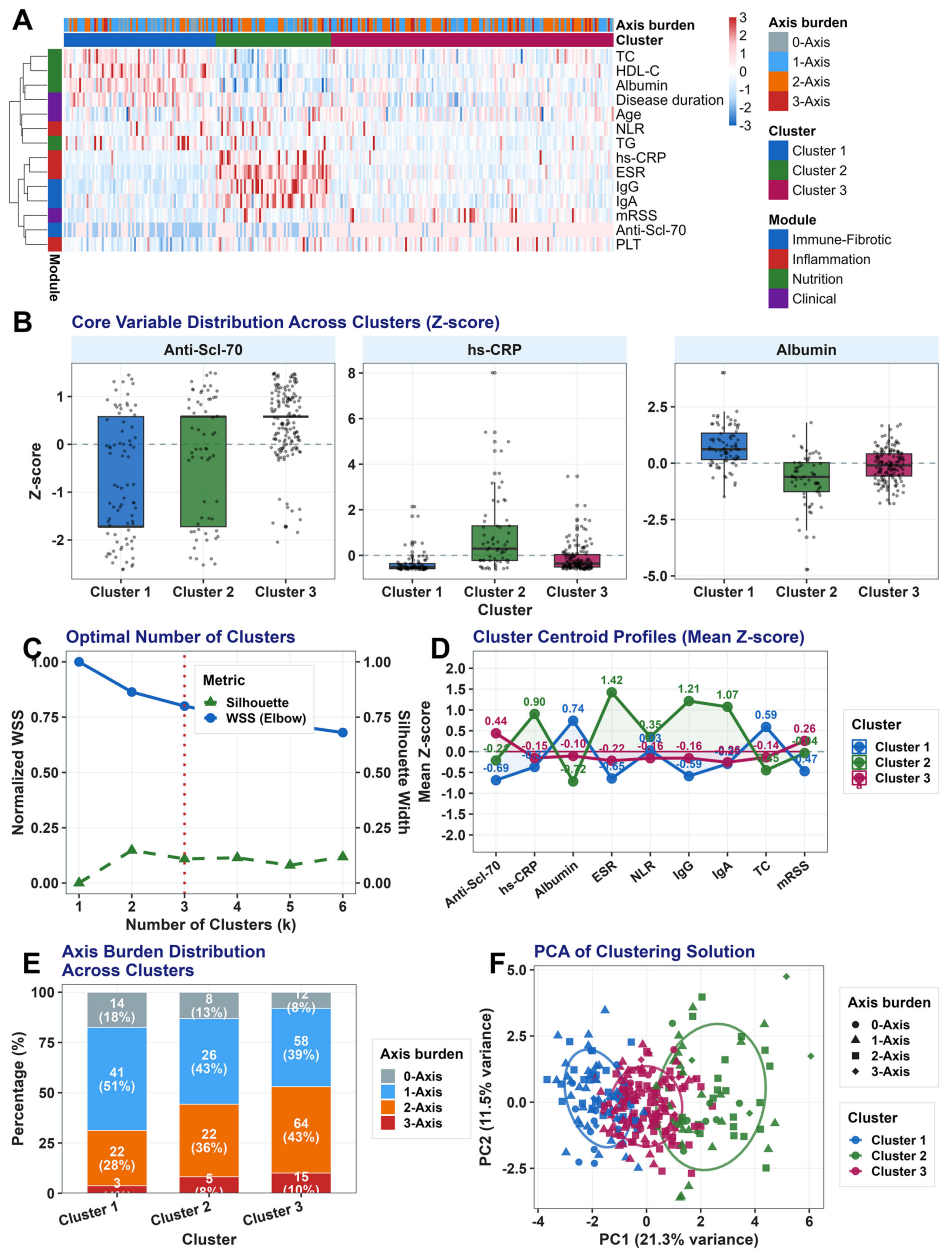
Unsupervised clustering identifies biomarker-defined subgroups with differing organ-burden distributions. **(A)** Heatmap of standardized core variables across individual patients, with annotations for axis burden, cluster assignment, and variable module. **(B)** Distribution of selected core variables across clusters, shown as Z-scores for Anti-Scl-70, hs-CRP, and albumin. **(C)** Selection of the optimal number of clusters using the elbow method and silhouette width. **(D)** Cluster centroid profiles shown as mean Z-scores for representative biomarkers and clinical variables. **(E)** Distribution of 0-Axis, 1-Axis, 2-Axis, and 3-Axis groups across clusters. **(F)** Principal component analysis (PCA) plot of the clustering solution, showing the separation and overlap among clusters and axis-burden groups. WSS, within-cluster sum of squares; PCA, principal component analysis.

Unsupervised clustering based on core variables identified a three-cluster solution (**Figure 3C**). The heatmap and centroid profiles showed differing biomarker patterns across clusters (**Figures 3A, D**). Cluster 1 showed relatively lower albumin and higher Anti-Scl-70 compared with some other clusters, Cluster 2 showed comparatively higher inflammatory features including hs-CRP and ESR, and Cluster 3 showed higher IgG and IgA together with intermediate inflammatory levels. Axis-burden distributions differed across clusters (**Figure 3E**). In Cluster 1, 14/80 (18%) were 0-Axis, 41/80 (51%) were 1-Axis, 22/80 (28%) were 2-Axis, and 3/80 (4%) were 3-Axis. In Cluster 2, 8/61 (13%) were 0-Axis, 26/61 (43%) were 1-Axis, 22/61 (36%) were 2-Axis, and 5/61 (8%) were 3-Axis. In Cluster 3, 12/149 (8%) were 0-Axis, 58/149 (39%) were 1-Axis, 64/149 (43%) were 2-Axis, and 15/149 (10%) were 3-Axis. PCA visualization showed partial separation among the three clusters, although overlap remained present (**Figure 3F**). As an exploratory complementary analysis, unsupervised clustering identified three biomarker-pattern clusters that were only partially concordant with the axis-burden groups, supporting structured heterogeneity while not replacing the clinically prespecified burden framework.

## Discussion

Systemic sclerosis is a multisystem disease in which skin fibrosis, interstitial lung disease, and gastrointestinal involvement often evolve together rather than as isolated events (1–4). Clinically, this co-occurrence matters because it influences disease burden, nutritional status, treatment exposure, and likely long-term outcomes (5–8). Pathobiologically, these manifestations arise within a shared environment of immune dysregulation, vascular injury, and fibrosis, but the relative contribution of each process may differ among patients (9, 10). In this retrospective cross-sectional study of 314 patients with SSc, we found that increasing skin–lung–gut co-involvement burden was accompanied by structured circulating biomarker patterning across clinically defined burden groups. Among the evaluated markers, Anti-Scl-70 positivity and lower albumin showed the most consistent associations with higher burden states in adjusted analyses, whereas hs-CRP differed across groups descriptively but showed less stable adjusted associations. Exploratory unsupervised clustering further suggested partially concordant biomarker-pattern heterogeneity beyond organ counting alone. Taken together, these findings support a clinically anchored and immunologically relevant view of heterogeneity in systemic sclerosis, while remaining firmly associative rather than mechanistic and complementary to conventional subtype-based classification (26). The principal signals identified here should also be interpreted as partly confirmatory of established subtype- and organ-involvement biology.

The association between Anti-Scl-70 positivity and higher burden groups is consistent with established knowledge of SSc subset biology. We therefore do not interpret this finding as a novel mechanistic discovery, but rather as evidence that the present pragmatic burden framework captures clinically meaningful disease structure. Anti-Scl-70 is strongly linked to diffuse cutaneous disease and higher risk of lung involvement (14, 15, 21). The graded increase in Anti-Scl-70 positivity across burden groups is notable and aligns with the established association of this autoantibody with more severe and fibrotic SSc phenotypes, particularly interstitial lung disease (27, 28). At the same time, our findings should not be interpreted as indicating that Anti-Scl-70 independently determines the full spectrum of organ co-involvement captured by the present framework. Rather, within this clinically defined burden structure, Anti-Scl-70 appears to mark a subgroup of patients with higher overall co-involvement load. Supplementary subtype-adjusted and subtype-stratified analyses supported the directional robustness of this signal, while also indicating overlap with established subtype-related biology (**Supplementary Figures 4, 5**).

The association between lower albumin and higher burden groups also deserves careful interpretation. Although albumin may reflect nutritional compromise in patients with substantial gastrointestinal or multisystem disease burden, it is not a nutrition-specific marker (29, 30). Albumin is also influenced by systemic inflammation, hepatic synthesis, vascular permeability, and other aspects of overall disease severity. Therefore, in the present study, lower albumin is best viewed as a clinically accessible integrative marker associated with higher organ co-involvement burden rather than as evidence of a single underlying biological pathway. It should not be interpreted as a disease-specific biomarker of SSc. Gastrointestinal involvement in SSc encompasses a heterogeneous spectrum of manifestations with differing relationships to nutritional status (31, 32). In our cohort, the internal composition of the gut-axis group is shown in **Supplementary Table 1**, and this heterogeneity should be considered when interpreting the albumin association. What is notable is that albumin remained one of the most stable adjusted associations even when immune and inflammatory variables were modeled together. This supports albumin as a practical integrative marker of higher burden in this specific framework (9).

Inflammatory markers were more nuanced. hs-CRP, PLT, and NLR differed across groups descriptively, and the 2-Axis and 3-Axis groups generally showed higher medians. However, after joint adjustment, hs-CRP was inversely associated with 1-Axis, but not with 2-Axis or 3-Axis. This pattern suggests that inflammatory signal intensity may vary across organ burden states, but that hs-CRP alone does not provide a stable discriminating marker for higher burden categories within this framework. Prior work has shown that inflammatory expression in SSc is heterogeneous and may differ between ILD-dominant and skin-dominant phenotypes (33, 34). One likely explanation is that the 1-Axis group was heavily lung-dominant, whereas the 2-Axis and 3-Axis groups incorporated more skin and gut co-involvement, producing a more complex biomarker structure than a single linear inflammatory pathway would capture. This interpretation was supported by the axis-specific supplementary analyses, in which the strongest adjusted signals were concentrated in the lung axis rather than being evenly distributed across all three organ domains (**Supplementary Figures 6A, B**). Another possibility is overlap between inflammatory and nutritional information, particularly between hs-CRP and albumin-related burden (35). The NLR and PLT showed descriptive increases across burden groups, consistent with reports of elevated NLR as a marker of immune activation and disease severity in SSc (36, 37). However, neither reached independent significance in the joint model, suggesting that their descriptive associations are partly mediated by or collinear with other immune and nutritional variables. The cholesterol finding in the 3-Axis group was unexpected and should be interpreted cautiously given the small sample size. Lipid metabolism in SSc show dyslipidemia may be related to treatment exposure and malnutrition (38, 39). Additional supplementary analyses incorporating treatment exposure and prednisone-equivalent glucocorticoid dose yielded directionally consistent results for the principal associations, although residual treatment confounding cannot be fully excluded (**Supplementary Figures 2, 7A, B**).

The clustering analysis adds a useful complementary perspective. Cluster 3 contained a higher proportion of 2-Axis and 3-Axis cases than Cluster 1, whereas Cluster 1 was relatively enriched for 1-Axis cases. This partial concordance supports the clinical relevance of the burden framework. At the same time, substantial overlap remained on PCA and the cluster distributions were not identical to the burden categories. This indicates that biomarker-defined structure is related to, but not reducible to, organ-count burden (40, 41). In practical terms, the burden framework captures one clinically useful layer of disease organization, while clustering suggests additional latent heterogeneity within and across burden groups. It should not be interpreted as defining mechanistic endotypes.

From a translational immunology perspective, the present study does not aim to define mechanistic endotypes, but rather to link clinically interpretable heterogeneity in a systemic autoimmune disease to immune-relevant circulating marker patterning. In this sense, the work is best viewed as clinically pragmatic and hypothesis-generating rather than mechanistically resolved. The study has several strengths. It uses a simple and clinically interpretable burden framework, includes multiple biomarker modules, and integrates conventional regression with unsupervised clustering. The main findings were internally coherent, with the strongest adjusted signals concentrated in Anti-Scl-70 and albumin.

This study has several limitations. First, its retrospective cross-sectional design precludes causal inference and does not capture temporal changes in organ burden or biomarker profiles. Second, this was a single-center study, which may limit generalizability to broader SSc populations. Third, the biomarker panel and the broad definition of gastrointestinal involvement were clinically pragmatic but relatively coarse, and objective GI investigations and standardized GI severity grading were not systematically available in structured form across the cohort, which may have reduced biological resolution. Fourth, the study is primarily descriptive and hypothesis-generating, without functional validation, longitudinal follow-up, or deeper immunological profiling. Future studies should validate this skin–lung–gut burden framework in multicenter prospective cohorts, incorporate longitudinal assessment, and test whether broader immune profiling can refine biomarker-informed stratification in SSc.

## Conclusions

Skin–lung–gut burden framework identified clinically distinct organ co-involvement groups in SSc that were accompanied by structured circulating biomarker patterning. The most consistent signals were higher Anti-Scl-70 positivity and lower albumin in higher burden groups, with supplementary analyses supporting robustness while clarifying the roles of subtype structure, lung-axis predominance, treatment exposure, and GI heterogeneity. These findings support a clinically anchored, biomarker-informed perspective on multisystem involvement in systemic sclerosis, but should be interpreted as associative and hypothesis-generating.

## Data Availability

The original contributions presented in the study are included in the article/**Supplementary Material**. Further inquiries can be directed to the corresponding author.
