# Supplementary material for: Biomarker patterns across skin–lung–gut burden in systemic sclerosis
Source: Front Immunol. 2026 Jul 17;17:1841944. doi: 10.3389/fimmu.2026.1841944 (PMC13439867; doi:10.3389/fimmu.2026.1841944)
Supplement: Supplementary file 1 [file Supplementaryfile1.pdf]

Supplementary materials

Supplementary Results

Sensitivity analyses for Anti-Scl-70, hs-CRP, and albumin

Sensitivity analyses were performed for Anti-Scl-70 positivity, hs-CRP, and albumin using the main joint multinomial model, an ordinal logistic model, and a joint multinomial model excluding sex as a covariate (Figure S1). For Anti-Scl-70 positivity, the main joint model showed OR 2.37 (95% CI 0.96–5.87, p=0.061) for 1-Axis versus 0-Axis, OR 3.86 (95% CI 1.46–10.22, p=0.007) for 2-Axis versus 0-Axis, and OR 12.78 (95% CI 1.86–87.91, p=0.010) for 3-Axis versus 0-Axis. In the ordinal model, Anti-Scl-70 positivity was associated with increasing burden (p=0.001). In the joint model without sex as a covariate, the pattern was similar, with p=0.007 for 2-Axis versus 0-Axis and p=0.010 for 3-Axis versus 0-Axis. For hs-CRP, the main joint model showed OR 0.60 (95% CI 0.37–0.98, p=0.039) for 1-Axis versus 0-Axis, OR 0.66 (95% CI 0.42–1.04, p=0.074) for 2-Axis versus 0-Axis, and OR 1.04 (95% CI 0.60–1.81, p=0.890) for 3-Axis versus 0-Axis. In the ordinal model, hs-CRP was not associated with increasing burden overall. In the joint model without sex as a covariate, the inverse association for 1-Axis versus 0-Axis remained (p=0.039), whereas the 2-Axis and 3-Axis comparisons remained non-significant. For albumin, the main joint model showed OR 0.62 (95% CI 0.37–1.03, p=0.067) for 1-Axis versus 0-Axis, OR 0.52 (95% CI 0.31–0.89, p=0.017) for 2-Axis versus 0-Axis, and OR 0.38 (95% CI 0.18–0.79, p=0.009) for 3-Axis versus 0-Axis. In the ordinal model, lower albumin was associated with increasing burden (Figure S1). In the model without sex as a covariate, the higher-burden associations were retained, with p=0.010 for 2-Axis versus 0-Axis and p=0.009 for 3-Axis versus 0-Axis. The sensitivity analyses were consistent for Anti-Scl-70 positivity and albumin, whereas hs-CRP showed a limited and non-monotonic pattern.

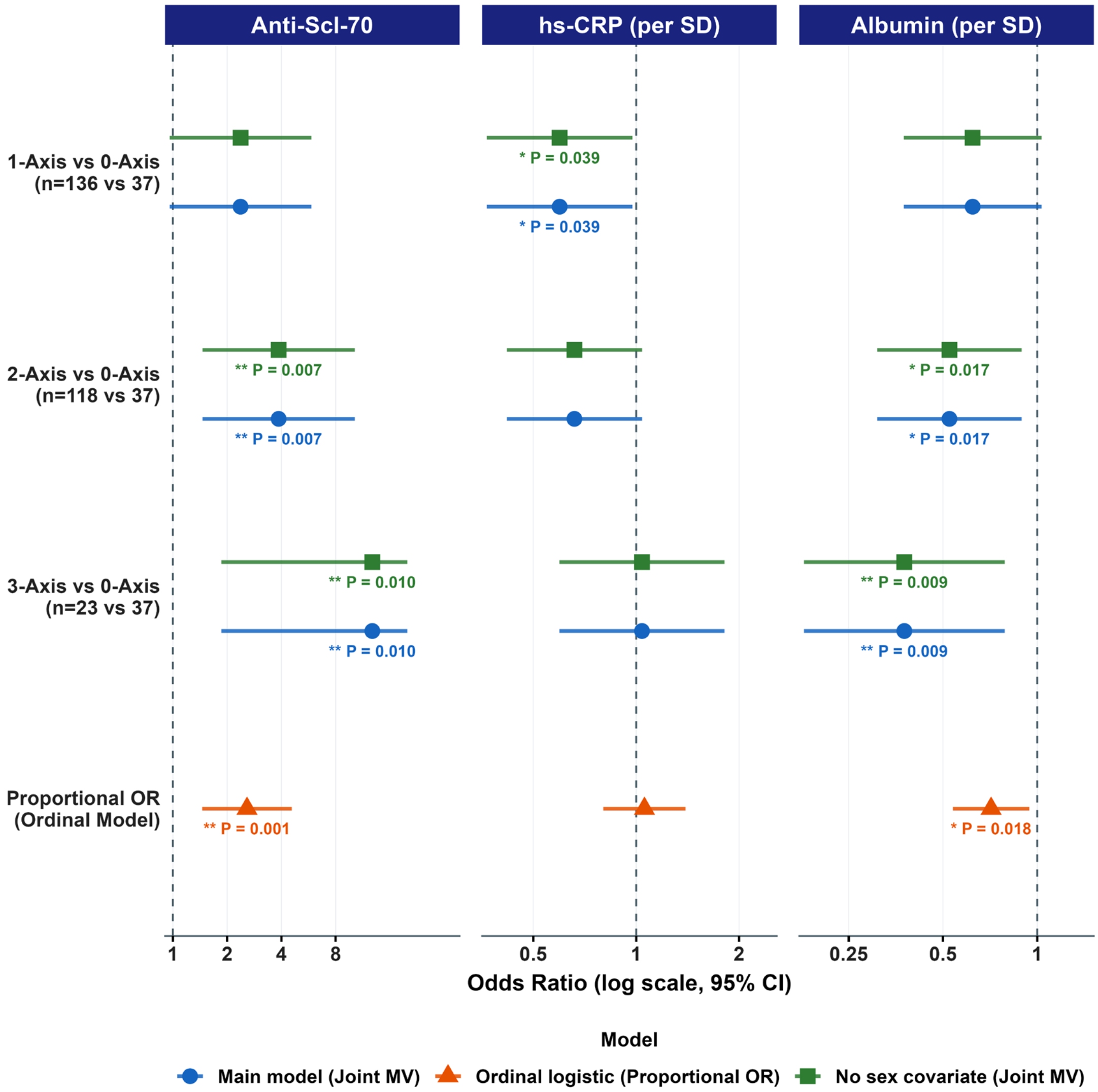

Supplementary Figure S1. Sensitivity forest plots for Anti-Scl-70 positivity, hs-CRP, and albumin across skin－lung–gut burden comparisons Forest plots showing odds ratios and 95% confidence intervals for Anti-Scl-70 positivity, hs-CRP, and albumin across 1-Axis versus 0-Axis, 2-Axis versus 0-Axis, and 3-Axis versus 0-Axis comparisons. Three model specifications are shown: the main multivariable joint multinomial model, an ordinal logistic model treating burden as an ordered outcome, and a joint multinomial model excluding sex as a covariate. *, P<0.05; **, P<0.01.

Sensitivity analyses with additional adjustment for treatment exposure

To assess the potential influence of treatment exposure, we performed treatment-adjusted sensitivity analyses using three nested multinomial logistic regression models.

For Anti-Scl-70 positivity, the odds ratios for 1-Axis vs 0-Axis were 2.56 (95%CI 1.19–5.50), 2.78(1.26–6.10), and 2.89 (1.30–6.42) across M1–M3, respectively. For albumin (per SD increase), the corresponding odds ratios were 0.76 (0.50 –1.15), 0.78 (0.51–1.20), and 0.73 (0.47–1.13) for 1-Axis vs 0-Axis; 0.58 (0.37–0.89), 0.59 (0.38–0.93), and 0.55(0.35–0.87) for 2-Axis vs 0-Axis; and 0.39 (0.22–0.69), 0.40 (0.22–0.72), and 0.37 (0.21–0.67) for 3-Axis vs 0-Axis, respectively. The effect estimates remained directionally and quantitatively similar after additional adjustment for glucocorticoid and immunosuppressant use, supporting the robustness of the main findings.

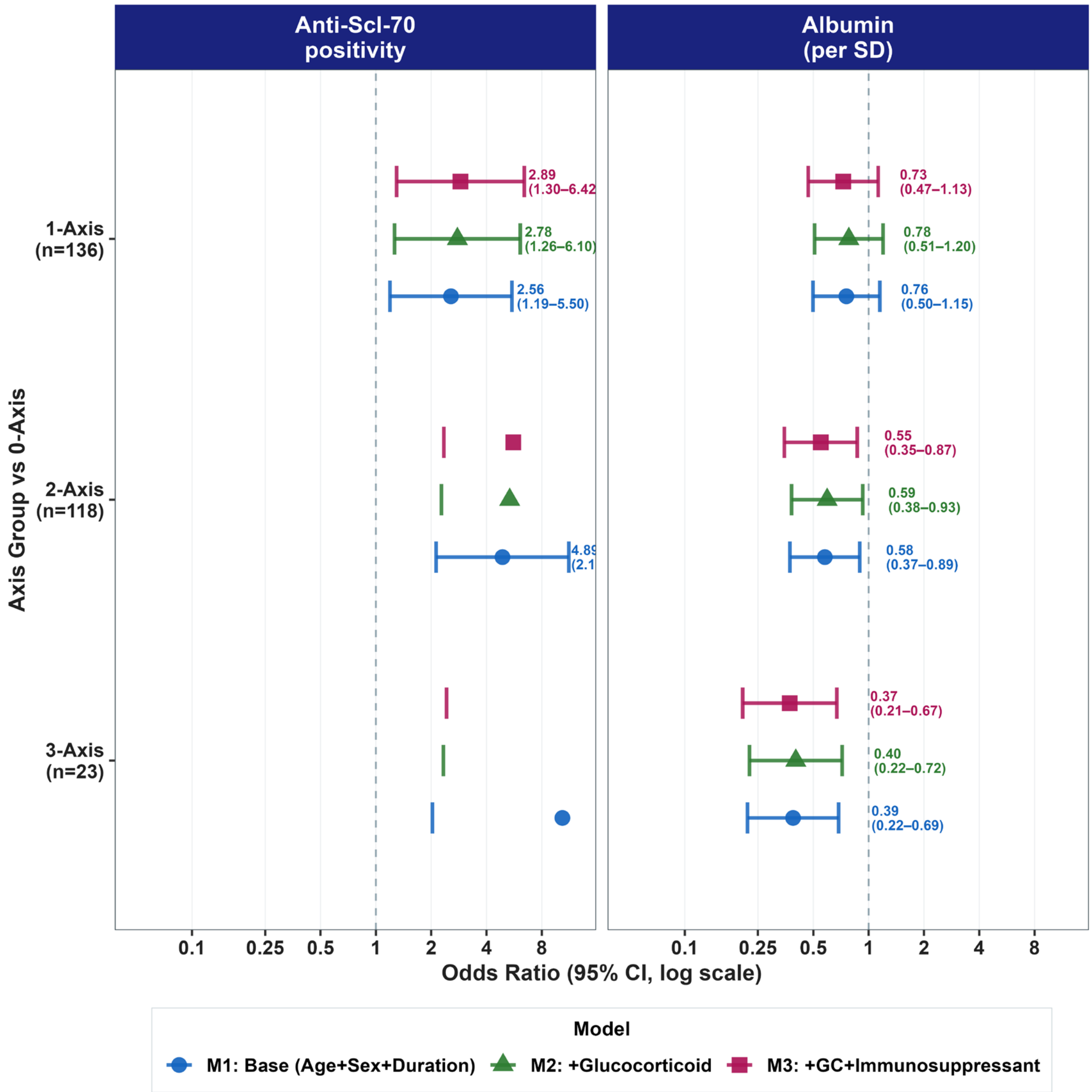

Supplementary Figure S2. Treatment-adjusted sensitivity analyses for the associations of Anti-Scl-70 positivity and albumin with organ-burden groups Forest plots show odds ratios (ORs) and 95% confidence intervals (CIs) from multinomial logistic regression models comparing the 1-Axis, 2-Axis, and 3-Axis groups with the 0-Axis group as reference.

Effect-size and significance overview in the joint multivariable model

Figure S3 summarizes effect size and significance for the joint multivariable model across all biomarkers. In the nominal significance display (Figure S3A), the most prominent signals were hs-CRP for 1-Axis versus 0-Axis (OR 0.60, 95% CI 0.37 – 0.98, p=0.039), Anti-Scl-70 positivity for 2-Axis versus 0-Axis (OR 3.86, 95% CI 1.46 – 10.22, p=0.007) and 3-Axis versus 0-Axis (OR 12.78, 95% CI 1.86 – 87.91, p=0.010), and albumin for 2-Axis versus 0-Axis (OR 0.52, 95% CI 0.31 – 0.89, p=0.017) and 3-Axis versus 0-Axis (OR 0.38, 95% CI 0.18 – 0.79, p=0.009). Total cholesterol was additionally associated with 3-Axis versus 0-Axis (OR 2.81, 95% CI 1.11 – 7.13, p=0.030). ACA for 3-Axis versus 0-Axis appeared as an extreme point because no ACA-positive cases were present in the 3-Axis group. In the FDR-adjusted display (Figure S3B), Anti-Scl-70 positivity for 2-Axis and 3-Axis, albumin for 2-Axis and 3-Axis, and hs-CRP for 1-Axis remained the most visually prominent findings, whereas most other biomarkers clustered near the null region with lower adjusted significance. The 3-Axis comparisons showed wider dispersion and lower precision, consistent with the smaller sample size of that group. The effect-size plots support Anti-Scl-70 positivity and lower albumin as the clearest adjusted signals, with hs-CRP contributing a narrower signal restricted mainly to the 1-Axis comparison.

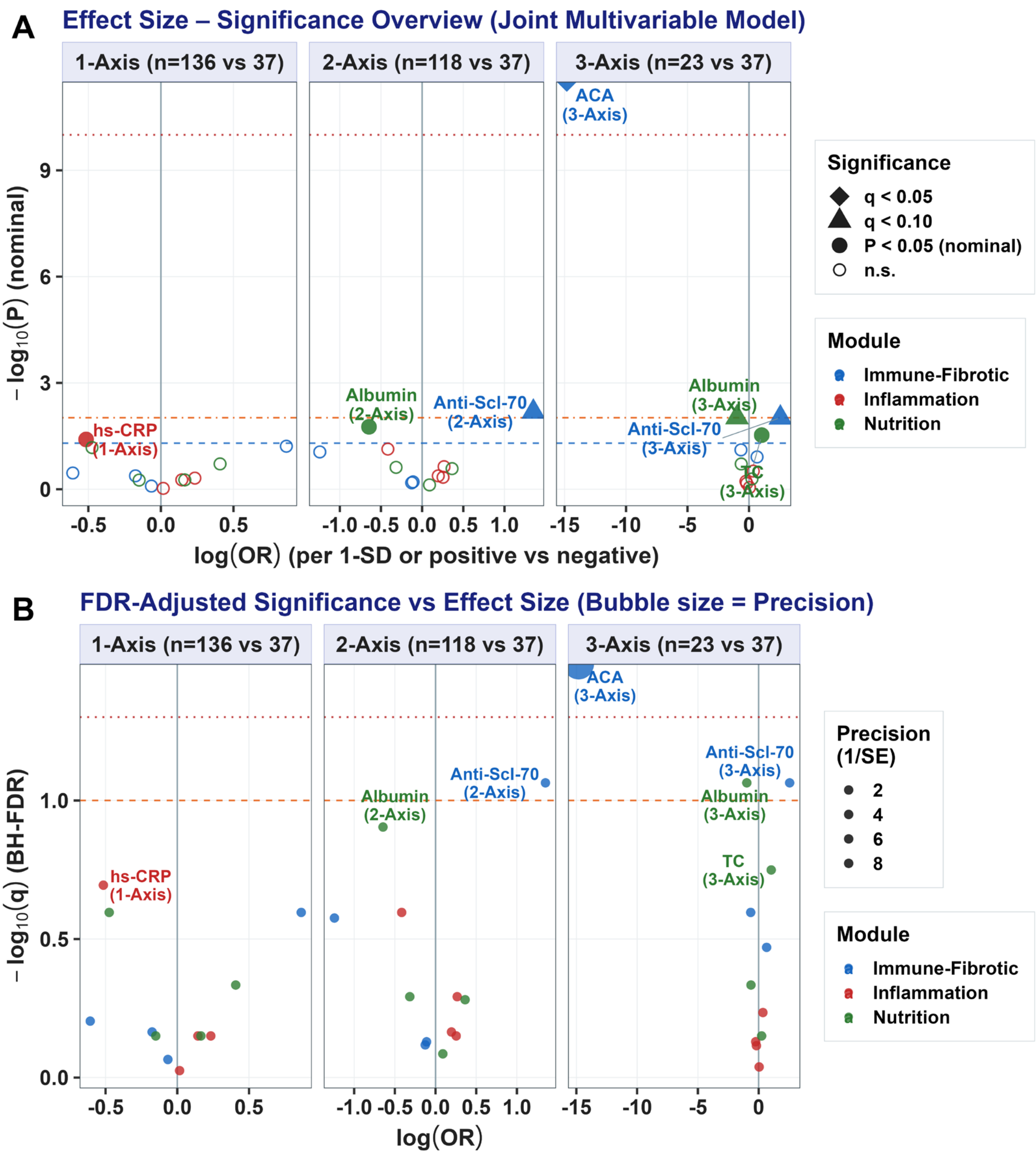

Supplementary Figure S3. Effect-size and significance overview for the multivariable joint model across burden comparisons (A) Nominal significance versus effect size. (B) FDR-adjusted significance versus effect size. Points are colored by module: immune-fibrotic, inflammation, and nutrition. The plots highlight Anti-Scl-70 positivity and albumin as the most prominent adjusted signals, while also showing limited precision for some 3-Axis estimates.

Subtype-adjusted multinomial logistic regression

To assess whether the principal biomarker associations persisted after accounting for conventional SSc subtype structure, we repeated the multinomial logistic regression analyses with additional adjustment for dcSSc/lcSSc subtype, while retaining age, sex, and disease duration as covariates (Figure S5). For clarity, this figure focuses on the higher-burden contrasts (2-Axis versus 0-Axis and 3-Axis versus 0-Axis).

After subtype adjustment, Anti-Scl-70 positivity remained the most prominent positive association with higher burden, particularly for 3-Axis versus 0-Axis, with a similar directional signal also present for 2-Axis versus 0-Axis. Albumin remained inversely associated with higher burden, again with the clearest signal in the higher-burden comparisons. ACA showed inverse associations, whereas PLT showed a positive association. By contrast, ESR, hs-CRP, NLR, IgG, IgA, TC, and TG did not show comparably strong or consistent higher-burden associations after subtype adjustment. These findings support the interpretation that the principal adjusted signals, especially Anti-Scl-70 and albumin, were not solely explained by dcSSc/lcSSc imbalance.

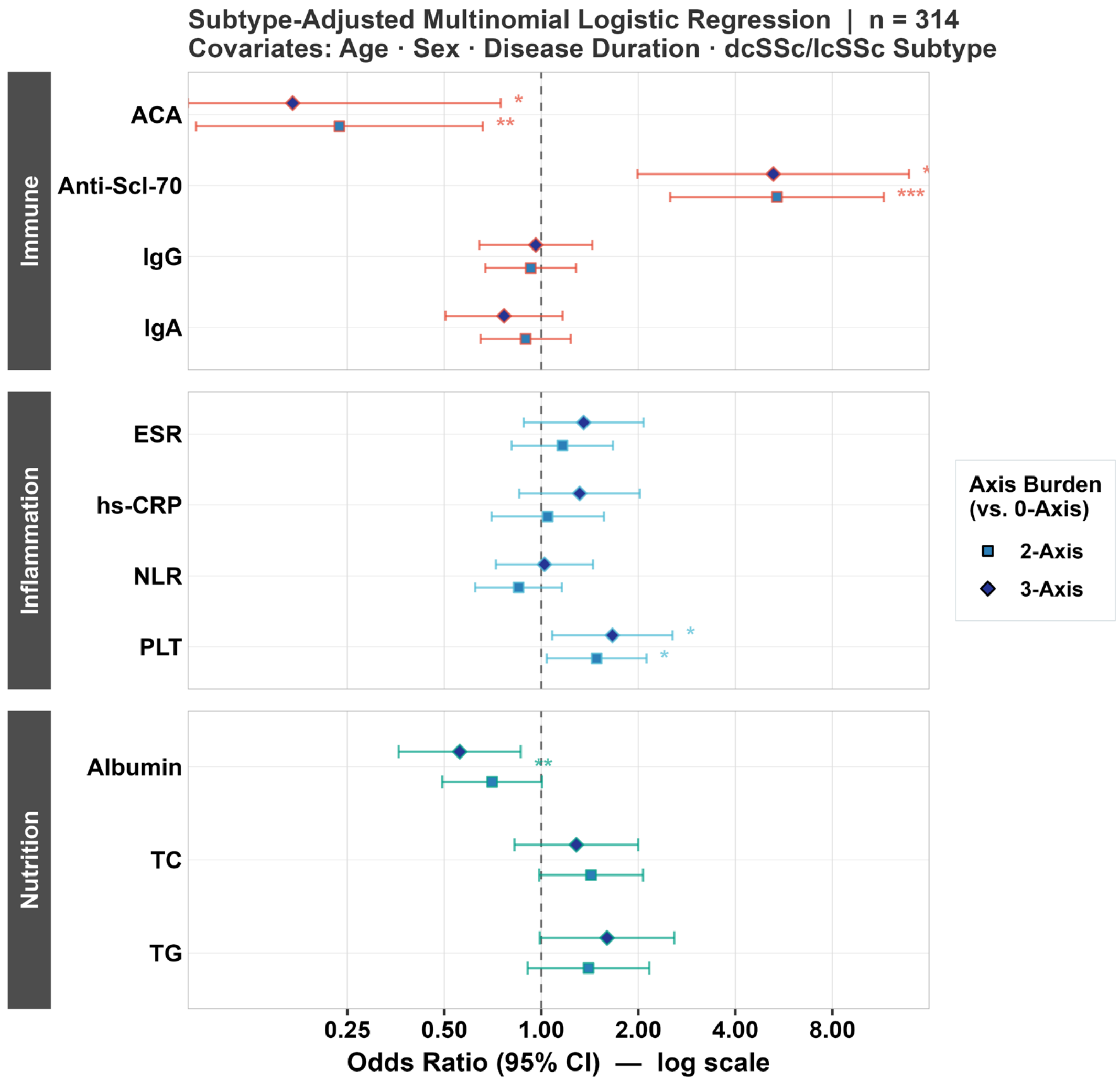

Supplementary Figure S4. Subtype-adjusted multinomial logistic regression across higher-burden comparisons Forest plot showing odds ratios (ORs) and 95% confidence intervals (CIs) from multinomial logistic regression models additionally adjusted for dcSSc/lcSSc subtype, with 0-Axis as the reference group. For clarity, only 2-Axis versus 0-Axis and 3-Axis versus 0-Axis are displayed. Continuous predictors are shown per 1-SD increase; ACA and Anti-Scl-70 are binary variables. Reference group: 0-Axis. Continuous predictors per 1-SD increase (z-scored); ACA & Anti-Scl-70 as binary (0/1). *, P<0.05; **, P<0.01; ***, P<0.001.

Subtype-stratified sensitivity analyses within dcSSc and lcSSc

To further evaluate whether the principal biomarker signals remained directionally consistent within conventional subtype strata, we performed subtype-stratified exploratory sensitivity analyses in the dcSSc and lcSSc subgroups (Figure S6). Because 0-Axis cases were absent within subtype strata, the 1-Axis group was used as the within-subtype reference category. Accordingly, the analyses compare 2-Axis versus 1-Axis and 3-Axis versus 1-Axis within each subtype.

Within the dcSSc subgroup, Anti-Scl-70 positivity remained positively associated with higher burden in both 2-Axis versus 1-Axis and 3-Axis versus 1-Axis comparisons. Albumin showed an inverse association, with lower albumin in the higher-burden groups relative to the 1-Axis group. hs-CRP showed a positive directional shift but without a comparably stable pattern. Within the lcSSc subgroup, the estimates were generally less precise and did not show clear significance, consistent with the smaller subgroup size and more limited within-stratum burden variation. Overall, these subtype-stratified analyses support directional consistency for Anti-Scl-70 and albumin, while indicating that some subtype-specific estimates should be interpreted cautiously.

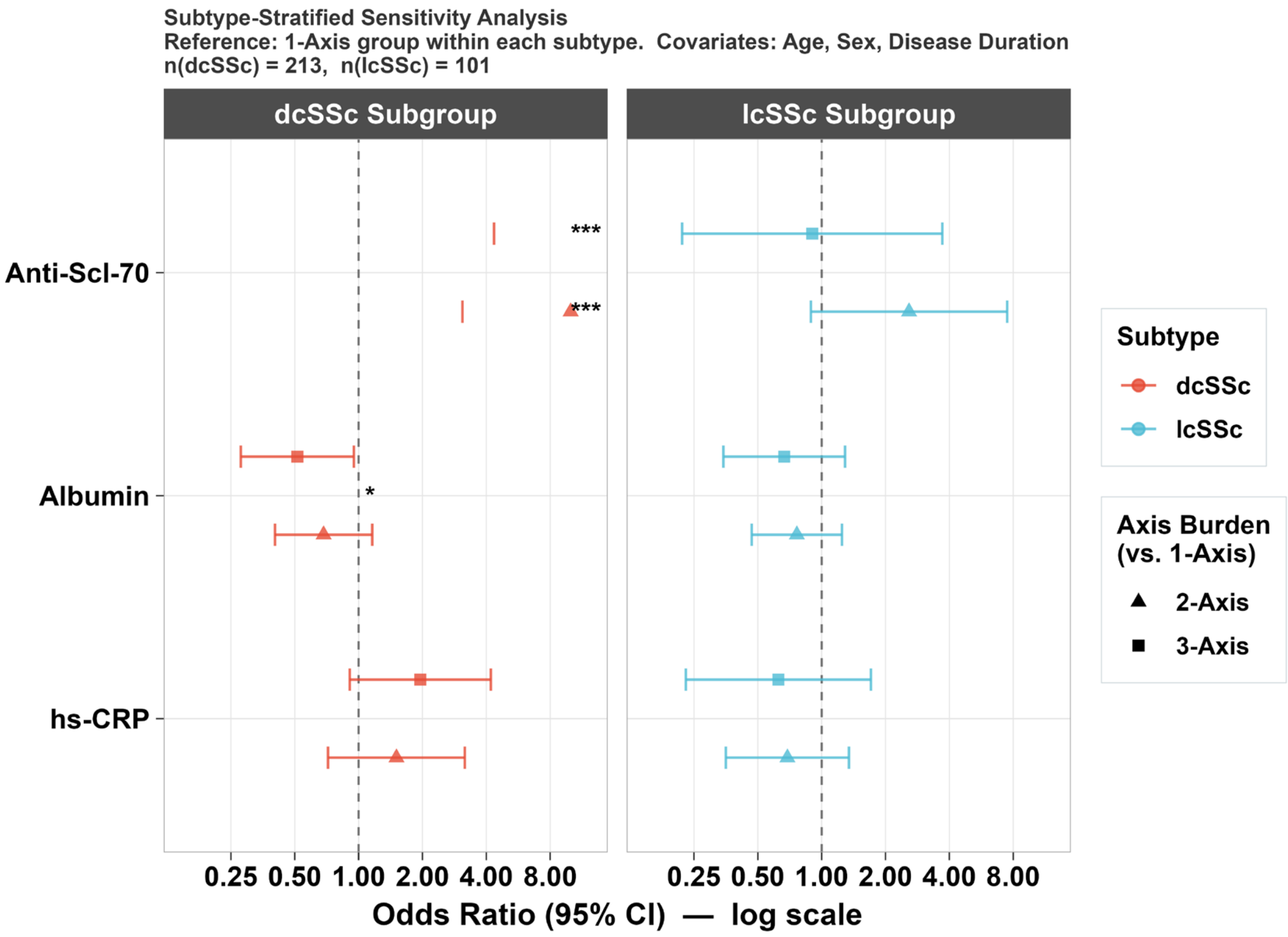

Supplementary Figure S5. Subtype-stratified sensitivity analyses within dcSSc and lcSSc Forest plots showing odds ratios (ORs) and 95% confidence intervals (CIs) for Anti-Scl-70, albumin, and hs-CRP in subtype-stratified multinomial logistic regression models. Analyses were adjusted for age, sex, and disease duration. The 1-Axis group was used as the within-subtype reference because 0-Axis cases were absent within subtype strata. The figure shows 2-Axis versus 1-Axis and 3-Axis versus 1-Axis comparisons; sparse 3-Axis estimates should be interpreted cautiously. OR per 1-SD increase (continuous) or per unit (Anti-Scl-70, binary). Reference group: 1-Axis (0-Axis absent within subtype strata). *, P<0.05; ***, P<0.001.

Axis-specific adjusted analyses independent of the burden-counting framework

Because the lower burden categories in this cohort were strongly shaped by lung-dominant composition, we performed an additional analysis examining each organ axis independently of the burden-counting framework (Figure S7). Specifically, the skin axis, lung axis, and gut axis were each analyzed separately as binary outcomes using adjusted logistic regression.

Among the primary biomarkers, Anti-Scl-70 showed the strongest positive association with lung-axis involvement ($p<0.001$), whereas the corresponding associations with the skin and gut axes were not significant. Albumin showed an inverse association with lung-axis involvement ($p=0.039$), whereas associations with the skin and gut axes were not significant. hs-CRP was not significantly associated with any single axis, although the lung-axis estimate showed a borderline directional trend ($p=0.06$). Among the secondary biomarkers, ACA was inversely associated with lung-axis involvement ($p=0.027$), and total cholesterol was positively associated with the lung axis ($p=0.020$), whereas the corresponding skin- and gut-axis estimates were not significant. These analyses indicate that the observed burden-related biomarker patterning was not uniformly distributed across all organ domains and that a substantial component of the signal was linked to lung-axis involvement.

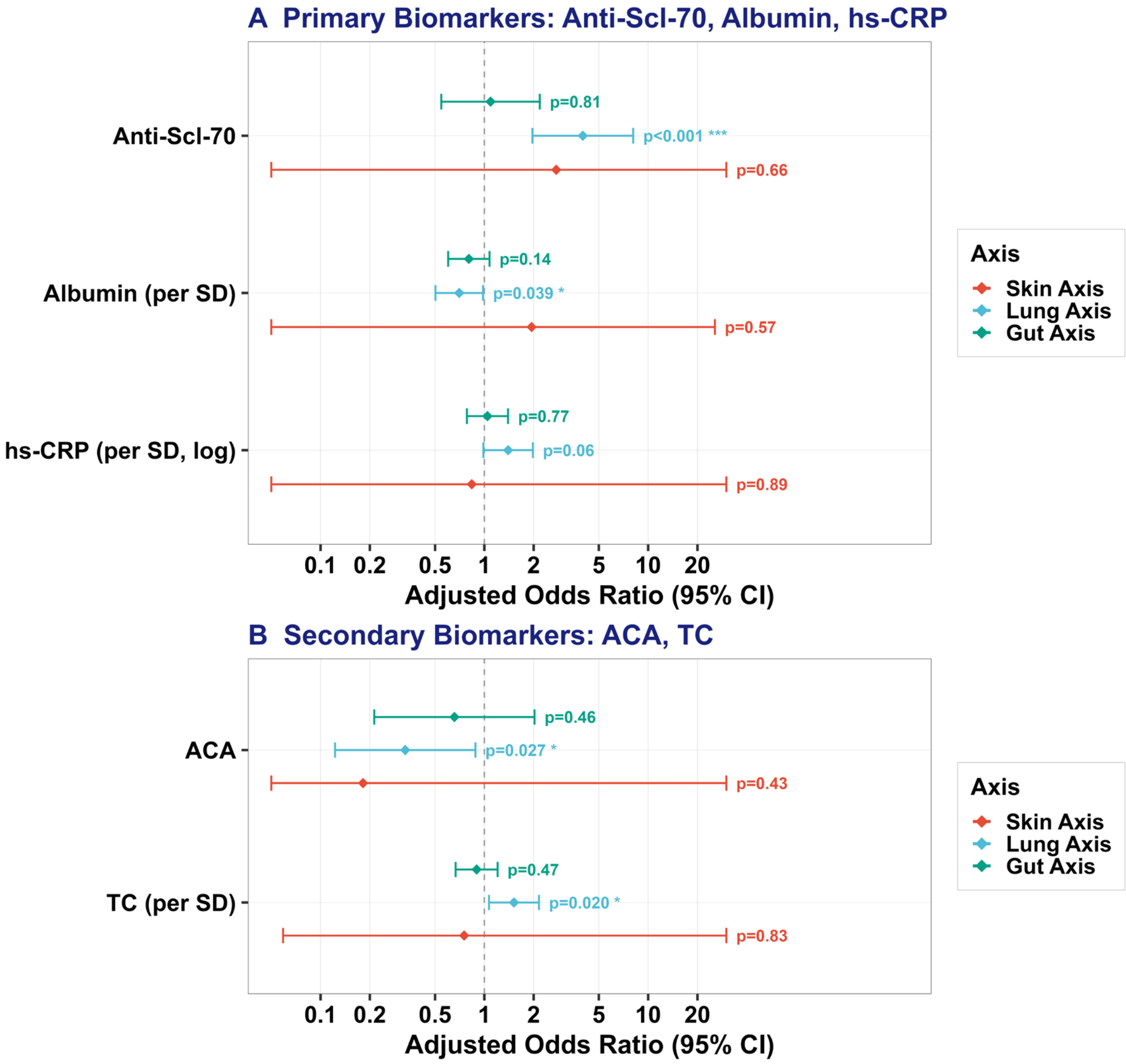

Supplementary Figure S6. Axis-specific adjusted logistic regression for skin-, lung-, and gut-axis involvement Adjusted logistic regression forest plots for biomarker associations with skin-axis, lung-axis, and gut-axis involvement analyzed separately as binary outcomes. Panel A shows Anti-Scl-70, albumin, and hs-CRP; Panel B shows ACA and total cholesterol. The reference group for each model was non-involvement of the corresponding axis. Anti-Scl-70 & ACA as binary (0/1); Albumin, hs-CRP, TC per 1-SD increase. Models were adjusted for age, sex, disease duration, and dcSSc/lcSSc subtype. *, P<0.05; ***, P<0.001.

Glucocorticoid dose distribution and dose-informed sensitivity analyses

Because albumin may be influenced by treatment exposure, particularly glucocorticoids, we further examined the distribution of prednisone-equivalent glucocorticoid dose across burden groups and performed dose-informed sensitivity analyses for the main biomarkers (Figure S9). Prednisone-equivalent dose data were available for 297 of 314 patients (94.6%). The median dose was 20 mg/day in each burden group, and the overall dose distribution did not differ significantly across groups (Kruskal–Wallis p=0.486). We then compared three nested multinomial logistic regression specifications for Anti-Scl-70, albumin, and hs-CRP: an unadjusted-for-GC model, a binary GC-adjusted model, and a dose-adjusted tertile model. For Anti-Scl-70, the estimates remained positive and directionally stable across all three models, with ORs of 2.36, 2.49, and 2.49 for 1-Axis versus 0-Axis; 3.66, 3.95, and 3.48 for 2-Axis versus 0-Axis; and 6.64, 7.90, and 7.06 for 3-Axis versus 0-Axis, respectively. For albumin, the inverse associations were likewise retained across models, with ORs of 0.76, 0.62, and 0.62 for 1-Axis versus 0-Axis; 0.60, 0.60, and 0.45 for 2-Axis versus 0-Axis; and 0.43, 0.43, and 0.45 for 3-Axis versus 0-Axis, respectively. hs-CRP showed a more limited pattern, with the inverse association concentrated mainly in the 1-Axis versus 0-Axis comparison. Overall, these findings suggest that the principal associations for Anti-Scl-70 and albumin were not materially altered by adjustment for glucocorticoid exposure, although residual treatment confounding cannot be fully excluded.

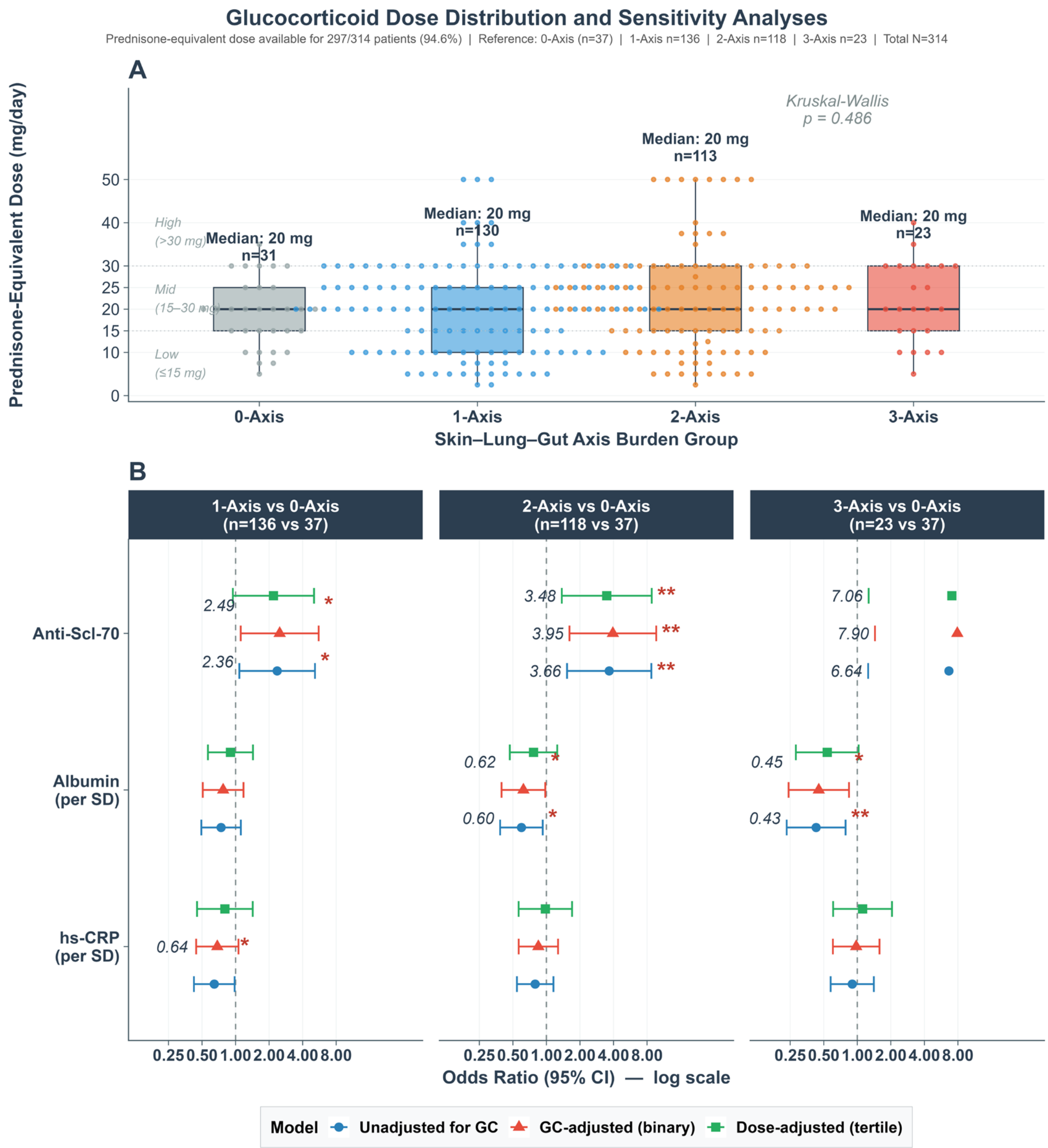

Supplementary Figure S7. Glucocorticoid dose distribution and dose-informed sensitivity analyses (A) The distribution of prednisone-equivalent glucocorticoid dose across burden groups. (B) Multinomial logistic regression estimates for Anti-Scl-70, albumin, and hs-CRP across 1-Axis, 2-Axis, and 3-Axis comparisons versus 0-Axis under three model specifications: unadjusted for glucocorticoids, binary GC-adjusted, and dose-adjusted (tertile). Anti-Scl-70 binary (0/1); Albumin & hs-CRP per 1-SD. Models were adjusted for age, sex (Female=1), disease duration, and diffuse SSc. *, P<0.05; **, P<0.01.

Composition of gastrointestinal manifestations within the gut-axis group

To clarify the internal composition of the gut axis, we summarized the distribution of chart-documented gastrointestinal manifestations within the 59 gut-axis-positive patients and across burden groups (Table S3). Within the gut-axis-positive group, dysphagia was the most frequent manifestation (16/59, 27.1%), followed by reflux/GERD (12/59, 20.3%), bloating/distension (9/59, 15.3%), and bowel involvement (6/59, 10.2%). Across the full cohort, the corresponding overall frequencies were 7.6% for dysphagia, 7.6% for reflux/GERD, 5.7% for bloating/distension, and 3.8% for bowel involvement.

Across burden groups, GI manifestations were concentrated mainly in the 2-Axis and 3-Axis categories, consistent with the structure of the gut-axis definition. Dysphagia was particularly enriched in the 3-Axis group (8/23, 34.8%), whereas reflux/GERD and bloating/distension were more broadly distributed across gut-axis-positive burden groups. These findings confirm that the gut axis in this cohort captured a heterogeneous cluster of upper- and lower-GI manifestations, rather than a single uniform GI phenotype. Because categories were not mutually exclusive, individual patients could contribute to more than one manifestation category. At the same time, objective GI investigations and standardized GI severity grading were not systematically available in structured form in the retrospective record and therefore could not be reliably quantified at the cohort level.

Supplementary Table S1. Composition of gastrointestinal manifestations within the gut-axis group and across axis burden groups (N=314)

GI Manifestation	Overall (N=314)	Within gut-axis+ (n=59)	0-Axis (n=37)	1-Axis (n=136)	2-Axis (n=118)	3-Axis (n=23)
Dysphagia	24 (7.6%)	16 (27.1%)	1 (2.7%)	4 (2.9%)	11 (9.3%)	8 (34.8%)
Reflux / GERD	24 (7.6%)	12 (20.3%)	2 (5.4%)	6 (4.4%)	13 (11.0%)	3 (13.0%)
Bloating / Distension	18 (5.7%)	9 (15.3%)	1 (2.7%)	5 (3.7%)	12 (10.2%)	0 (0.0%)
Bowel Involvement (diarrhea, constipation, pseudo-obstruction)	12 (3.8%)	6 (10.2%)	0 (0.0%)	5 (3.7%)	6 (5.1%)	1 (4.3%)

Data are presented as n (%). Axis definitions identical to the main manuscript (Figure 1-4 and Table 1-4): skin axis = mRSS >= 14; lung axis = ILD confirmed by HRCT; gut axis = bGI >= 1. The 'Within gut-axis+' column reports the proportion of each GI manifestation type among the 59 gut-axis positive patients (18.8% of total cohort, N=314), directly addressing the composition of the gut-axis group as requested by reviewers. GI manifestation data extracted from admission records and matched by admission number. Categories were not mutually exclusive. Objective GI investigations and standardized severity grading were not uniformly available (retrospective chart-based dataset).
